# Antibacterial Activity of *Boswellia sacra* Flueck. Oleoresin Extract against *Porphyromonas gingivalis* Periodontal Pathogen

**DOI:** 10.3390/antibiotics10070859

**Published:** 2021-07-15

**Authors:** Nashwah G. M. Attallah, Walaa A. Negm, Engy Elekhnawy, Najla Altwaijry, Elshaymaa I. Elmongy, Thanaa A. El-Masry, Eman A. Alturki, Doaa A. Yousef, Malak Y. Shoukheba

**Affiliations:** 1 Pharmaceutical Sciences Department, College of Pharmacy, Princess Nourah bint Abdulrahman University, Riyadh 84428, Saudi Arabia; ngmohamed@pnu.edu.sa (N.G.M.A.); naaltwaijry@pnu.edu.sa (N.A.); 2 Egyptian Drug Authority (EDA) (Previously NODCAR), Giza 8655, Egypt; 3 Pharmacognosy Department, Faculty of Pharmacy, Tanta University, Tanta 31527, Egypt; 4 Pharmaceutical Microbiology Department, Faculty of Pharmacy, Tanta University, Tanta 31527, Egypt; 5 Pharmaceutical Chemistry Department, Faculty of Pharmacy, Helwan University, Helwan 11795, Egypt; 6 Pharmacology Department, Faculty of Pharmacy, Tanta University, Tanta 31527, Egypt; thanaa.elmasri@pharm.tanta.edu.eg; 7 Pharmacognosy Department, College of Pharmacy, King Saud University, Riyadh 84428, Saudi Arabia; emalturki@ksu.edu.sa; 8 Oral Medicine, Periodontology, Oral Diagnosis, and Radiology Department, Faculty of Dentistry, Tanta University, Tanta 31527, Egypt; doaa.bayoumi@dent.tanta.edu.eg (D.A.Y.); malak.mohamed@dent.tanta.edu.eg (M.Y.S.)

**Keywords:** biofilm, chronic periodontitis, extracellular polysaccharides, GC-MS, qRT-PCR, scanning electron microscope

## Abstract

*Boswellia sacra* Flueck. oleoresin extract (frankincense) has traditionally been used in the treatment of different diseases, but there are no sufficient studies on its potential activity against periodontal pathogens. Therefore, antibacterial and antibiofilm activity of frankincense extract against *Porphyromonas gingivalis* clinical isolates were studied. The phytochemical composition of the volatile components of the extract was identified by GC-MS analysis revealing 49 compounds as *trans*-nerolidyl formate, cycloartenol acetate, ursenoic acid 3-oxomethyl ester, bisabolene epoxide, and kaur-16-ene. It decreased the growth and increased the leakage of nucleotides in 58.3% and 33.3% of isolates, respectively. Additionally, it reduced the extracellular polysaccharide production and the cell surface hydrophobicity in 41.67% and 50% of the isolates, respectively. Crystal violet assay revealed inhibition of biofilm formation by the tested isolates. Light microscope and scanning electron microscope were used to examine the biofilms and they confirmed the reduction of biofilm formation by frankincense extract. Downregulation of the genes linked to biofilm formation (*fim*A, *hag*A, and *hag*B) was observed using qRT-PCR after treatment with the frankincense extract. This study suggested that the frankincense extract could exhibit antibacterial and antibiofilm activity against *P. gingivalis* isolates. Thus, the frankincense extract could be used as a treatment approach for periodontitis.

## 1. Introduction

Pathogenic bacteria have many virulence features, such as enzymes, toxins, fimbriae, and capsules that decrease the host response via producing tissue breakdown, impeding tissue healing and weakening the host defense [1,2]. Chronic periodontitis (CP) is an inflammatory condition involving the destruction of the gingival tissues of the oral cavity. The pathogenesis of CP encompasses a complex interaction between the periodontal pathogens with the host immunity [3]. One of the major etiologic pathogens that contributes to CP is *Prophyromonus* *gingivalis*. It is a Gram-negative anaerobic bacterium that in addition to other pathogens composes the “red complex,” which is a prototype poly-bacterial pathogenic consortium in chronic periodontitis [4,5]. *P. gingivalis* can produce various virulence factors necessary for the colonization of the subgingival sites, circumventing the immune system and tissue destruction [6]. One of the important virulence factors of *P. gingivalis* is the type I fimbriae (FimA) which could be a mediator for both biofilm formation and adherence to epithelial cells of the gingiva. Additionally, it can produce an inflammatory response [7]. Gingipains, causing the proteolytic activity of *P. gingivalis*, are encoded mainly by Arg-gingipain A (*rgp*A) and Lys-gingipain (*kgp*) [8]. Gingipains are involved in the destruction of the periodontal tissues through the breakdown of the proteins responsible for the cellular adhesion to epithelial and endothelial cells. This proteolytic activity could result in cell death with compromising the tissue integrity and enhanced bacterial spreading [9].

The efficacy of scaling and root planning (SRP), as a part of the nonsurgical periodontal treatment, has been established through several longitudinal studies [10,11,12]. It is established that SRP can give good results in reducing the burden of etiologic microbes for several weeks [13]. However, recolonization of pathogenic bacteria that reside deeper in the pocket or invaded tissues occurs within weeks toward pretreatment levels [14]. Hence, the need to use local antiseptics or systemic antibiotic therapy is recommended to improve the treatment outcome [15]. Systemic antibiotics are of great benefit as they reach the periodontal tissues via blood to suppress periodontal pathogens [16]. However, the emergence of antibiotic-resistant bacteria has made infectious diseases more difficult to treat [17]. This issue has forced researchers to constantly search for novel effective antimicrobial agents against different pathogenic bacteria. These antimicrobial agents could work via disrupting bacterial virulence such as biofilm formation [18]. Bacterial pathogenicity is defined as its ability to cause disease. The degree of the bacterial pathogenicity depends mainly on the virulence factors of bacteria. Virulence factors help the bacteria overcome the various host defense systems. Nevertheless, the development of antibiotic resistance enables pathogenic bacteria to overcome antibiotics and to survive in the host [19]. Many plants are considered a valuable source for both traditional and modern medicine. Many people depend on medicinal plant products to maintain their health or treat diseases [20,21]. Frankincense, one of these interesting medicinal plant products and known as al-luban or olibanum is a resin gummy exudate obtained from the tree bark of different *Boswellia* sp. (*Burseraceae* family) such as *B. frereana, B. serrata, B. sacra, B. carterii, B. thurifera*, and *B. neglecta* [22]. It is used as a traditional therapy in some countries to treat different diseases [23,24,25]. In Oman, *B. sacra* gum extracts have long been used in folk medicine to strengthen teeth and gums, stimulate digestion, prevent halitosis, and treat a variety of other ailments, including inflammatory illnesses and cancer. frankincense is popularly used in traditional Chinese medicine to improve blood circulation and relieve discomfort in leprosy, gonorrhea, and cancer patients [26]. In Egypt, Frankincense was used in inflammatory disorders, tumors, oedema, bronchitis, and asthma [27]. While in Somalia and Ethiopia, olibanum is used to treat allergies, snake and insect bites, colds, coughs, diarrhea, headaches, syphilis, stomach complaints, back complaints, disinfections, purifications, and hygiene, as well as to correct female disorders during menopause and topically to treat wounds in humans and livestock [28]. In the recent decade, the use of olibanum for the treatment of numerous chronic inflammatory issues such as arthritis, chronic bowel illness, asthma, peritumoral cerebral edema, and other ailments has gained popularity in European countries [29]. However, there are no sufficient detailed studies on the potential activity of this resin gummy exudate against the periodontal pathogens [30].

This study aimed to investigate the antibacterial and antibiofilm activity of *Boswellia sacra* Flueck. oleoresin extract against the periodontal pathogen, *P. gingivalis*, and exploring the minimum inhibitory concentration (MIC) that could be used while treating periodontal infections. To the best of our knowledge, this is the study of the antimicrobial and antibiofilm activities of frankincense oleoresin extract against *P. gingivalis* clinical isolates [30].

## 2. Results

### 2.1. Gas Chromatography-Mass Spectrometry (GC-MS) Analysis

GC-MS analysis revealed 49 compounds from the frankincense oleoresin extract. The results are listed in Table 1 and presented in Figure 1. The retention times and mass spectra comparisons with the MS library were used to identify the sample. Mass fragmentation patterns of all detected compounds were displayed in Supporting Materials Figures S1–S49.

### 2.2. Antibacterial Activity of the Frankincense Oleoresin Extract

The frankincense oleoresin extract displayed antibacterial effect on the isolated *P. gingivalis* bacteria (*n* = 12) using the agar diffusion method. The range of the minimum inhibitory concentration (MIC) values of the extract, determined by the broth microdilution method, ranged from 500 to 1000 µg/mL as shown in Table 2.

### 2.3. Bacterial Growth Curve

**Figure 2:**
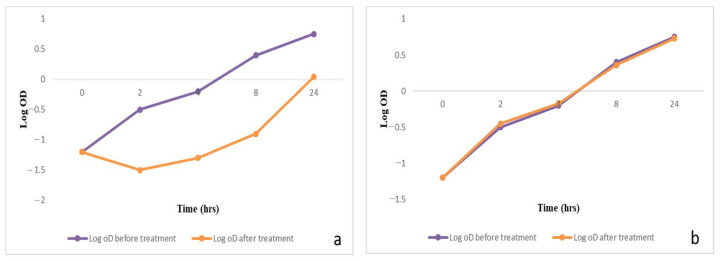
A graph showing *P. gingivalis* growth curves: (**a**) a significant decrease in bacterial growth after treatment with the frankincense extract (0.5 MIC); (**b**) non-significant change in the growth curve after treatment with 10%DMSO.

To evaluate the impact of the frankincense oleoresin extract on the growth process of *P. gingivalis* isolates, growth curves were created for the tested cells before and after the treatment of the frankincense oleoresin extract (0.5 MIC). A significant decrease (*p* < 0.05) in the growth of 58.3% of the tested isolates was observed and an illustrative example is shown in Figure 2. The remaining isolates showed a non-significant change (*p* > 0.05) after treatment.

### 2.4. Measurement of Nucleic Acid Leakage

**Figure 3:**
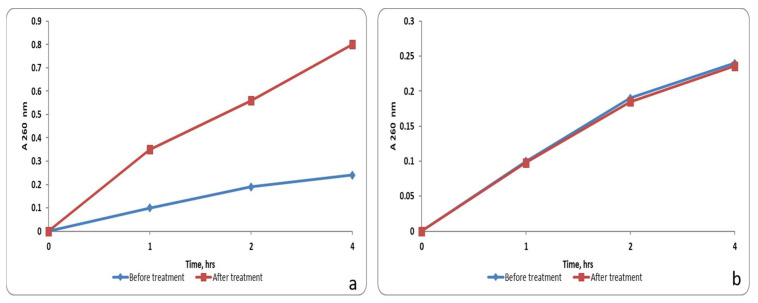
A graph showing the nucleic acid leakage from *P. gingivalis* isolates: (**a**) a significant increase in nucleic acid leakage after treatment with the frankincense extract (0.5 MIC); (**b**) non-significant change in nucleic acid leakage after treatment with 10% DMSO.

We observed that the frankincense extract (0.5 MIC) increased the release of nucleotides from intracellular compartments significantly (*p* < 0.05) in 33.3% of the tested isolates by measuring the absorbance at 260 nm using an 1800 UV-VIS spectrophotometer. A representative example is shown in Figure 3. The remaining isolates showed a non-significant change (*p* > 0.05) after treatment.

### 2.5. Bacterial Cell Surface Hydrophobicity

**Figure 4:**
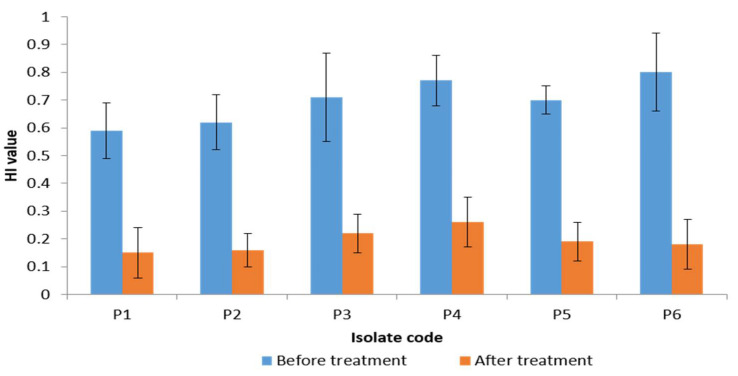
A chart showing a significant reduction of hydrophobicity index (HI) of 6 *P. gingivalis* isolates (50% of isolates) after treatment with the frankincense extract.

The cell surface hydrophobicity was investigated in the *P. gingivalis* isolates, before and after treatment with the frankincense extract via measuring of hydrophobicity index (HI). The bacterial surface hydrophobicity is an important factor contributing to bacterial adherence and biofilm formation. We found that there was a significant decrease in the HI in 50% of the tested isolates after treatment as shown in Figure 4. The remaining isolates showed a non-significant change in HI (*p* > 0.05) after treatment.

### 2.6. Antibiofilm Activity of Frankincense Extract

**Figure 5:**
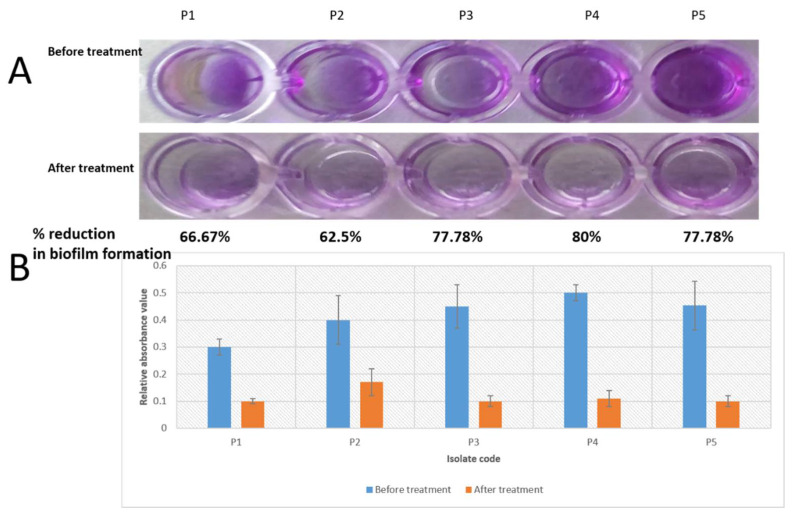
A significant reduction of biofilm formation by five *P. gingivalis* isolates induced by the frankincense extract: (**A**) crystal violet assay in microtitration plate; (**B**) a chart representing the significant decrease in the relative absorbance values of the formed biofilms after treatment.

Crystal violet assay was utilized to assess the production of mature biofilms by *P. gingivalis* isolates in 96-well microtitration plates. We noted that the frankincense extract significantly (*p* < 0.05) inhibited the biofilm formation in five *P. gingivalis* isolates (41.67% of isolates) with percentages of biofilm reduction that ranged from (62.5–80%) as shown in Figure 5. The remaining isolates showed a non-significant change (*p* > 0.05) in biofilm formation after treatment.

### 2.7. Extracellular Polysaccharide Measurement

**Figure 6:**
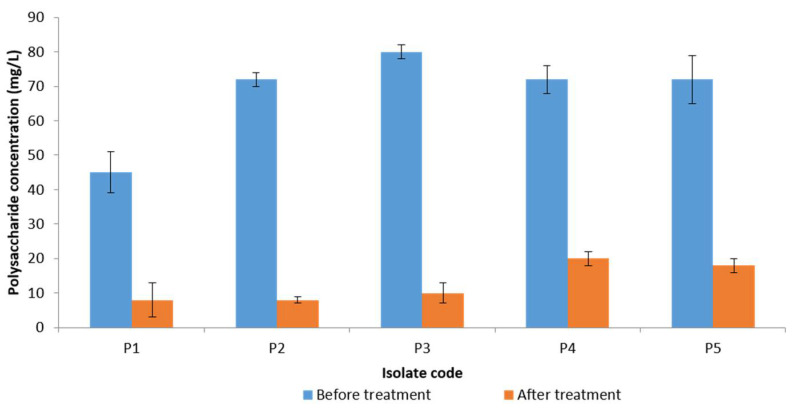
A chart showing a significant reduction of extracellular polysaccharide production by five *P. gingivalis* isolates after treatment with the frankincense extract.

The extracellular polysaccharides, which are the main components of the biofilms, were assayed before and after treatment with the frankincense extract using the phenol–sulfuric acid method. A significant reduction (*p* < 0.05) of the quantity of the extracellular polysaccharides was observed in five *P. gingivalis* isolates (which showed inhibition of their biofilm formation by crystal violet assay) as shown in Figure 6. The remaining isolates showed a non-significant change (*p* > 0.05) after treatment.

### 2.8. Examination of the Biofilm Morphology by Light Microscope and Scanning Electron Microscope (SEM)

**Figure 7:**
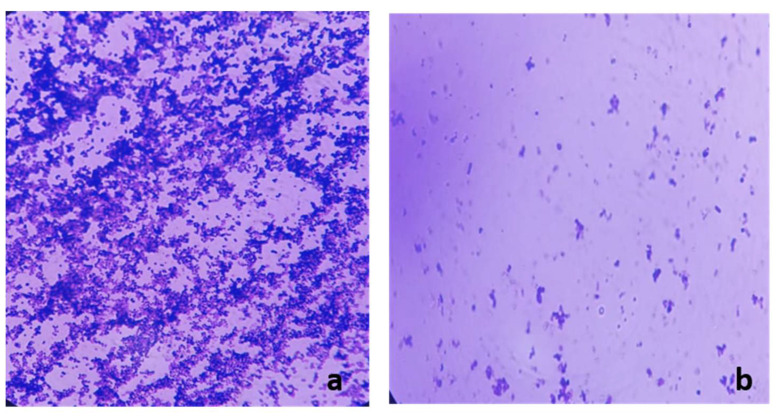
A representative example for the significant reduction of biofilm formation by *P. gingivalis* isolates examined by light microscope after staining with crystal violet: (**a**) before treatment and (**b**) after treatment with the frankincense extract.

Light microscope analysis of the formed biofilms before and after treatment with frankincense extract confirmed the inhibition of biofilm formation in five *P. gingivalis* isolates which were found to be inhibited by semi-quantitative crystal violet assay. Visible reductions of biofilms on the treated slides were noted after crystal violet staining as shown in Figure 7.

**Figure 8:**
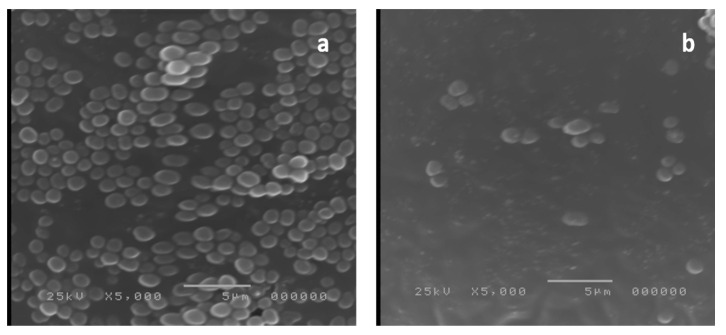
A representative example for the significant reduction of biofilm formation by *P. gingivalis* isolates examined by scanning electron microscope: (**a**) before treatment and (**b**) after treatment with the frankincense extract.

It is well known that electron microscopes could produce images of the tested specimens with higher resolution than those produced by light microscopes. Therefore, we examined five *P. gingivalis* isolates, previously examined by light microscope, using SEM. The SEM examination demonstrated a significant reduction in the aggregation of *P. gingivalis* bacterial cells in the formed biofilms after treatment with frankincense extract as shown in Figure 8.

### 2.9. Quantitative RT-PCR

qRT-PCR was utilized to study the impact of frankincense extract on the *P. gingivalis* virulence factors which are related to biofilm formation. As shown in Table 3, the gene expression of *fim*A, *hag*A, and *hag*B was found to be significantly decreased in 50%, 41.7%, and 50% of the isolates, respectively. The remaining isolates showed a non-significant change in the gene expression (*p* > 0.05) after treatment. In addition, there was a non-significant change in the gene expression of *rgp*A and *kgp* in all isolates.

### 2.10. Cytotoxicity Assay

The cytotoxic effect of *Boswellia sacra* Flueck. oleoresin extract in a human skin fibroblast (HSF) cell line was determined using the SRB (sulforhodamin B) assay [31]. The results exhibited that the frankincense extract at the concentration of ≤30 µg/mL displayed no cytotoxicity, whereas that at >30–300 µg/mL exhibited mild cytotoxicity in the HSF cell line with IC_50_ = 30.7 ± 4.41 µg/mL in comparison to doxorubicin as a positive control IC_50_ = 4.36 ± 0.52 µg/mL.

## 3. Discussion

**Figure 9:**
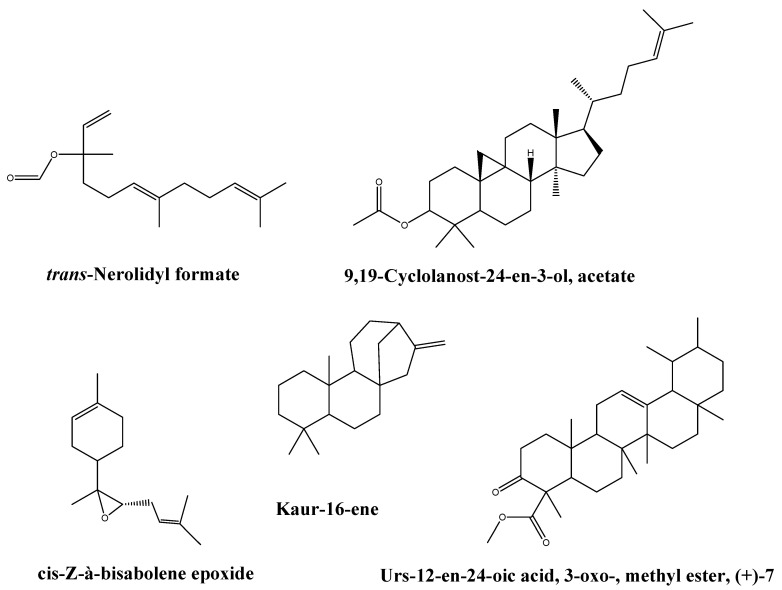
The chemical structure of the main identified compounds from the *B. Sacra* oleoresin extract.

Considering the vast spread of bacterial resistance to different antibiotics, which is now becoming an increasing worldwide health threat, researchers are seeking alternative strategies to prevent or limit bacterial infections. Recently, there has been growing evidence demonstrating that the plant extracts could offer promising antimicrobial and antibiofilm potentials with no risk of increasing resistance to antibiotics. A great number of phytochemicals have been documented to be valuable alternatives and supplementary products that could be used in our battle against bacterial infections [32,33]. The frankincense extract reveals a wide variety of different classes of active compounds such as steroids, sesquiterpenes, hormones, vitamins, oxygenated sesquiterpenes, pentacyclic, tetracyclic triterpenes, fatty acids, and other polycyclic aromatic hydrocarbons. Among 49 compounds detected by GC/MS of the frankincense oleoresin extract, *trans*-Nerolidyl formate was the main compound representing 19.88% followed by cycloartenol acetate, (3á)-, urs-12-en-24-oic acid, 3-oxo-, methyl ester, (+)-7, cis-Z-à-bisabolene epoxide, and kaur-16-ene representing 13.56%, 12.42%, 9.41%, and 7.46% of total extract, respectively. *Trans*-nerolidyl formate belongs to sesquiterpene, kaur-16-ene belongs to diterpenes. While from triterpenes, cycloartenol acetate, (3á)-, urs-12-en-24-oic acid, 3-oxo-, methyl ester, (+)-7, and cis-Z-à-bisabolene epoxide were identified. Fragmentation patterns of these main compounds are presented in Figures S15, S43, S37, S16, and S9, while the chemical structures are represented in Figure 9. This is the first report for the identification of these compounds from the frankincense (*B. Sacra*) extract.

The activity of frankincense extract could be attributed to the variety of active compounds detected in it, especially *trans*-Nerolidyl formate which exhibited antimicrobial activity at 30 μM against airborne microbes and inhibited the growth of *P. obscurans* by 49.9% in comparison with the control (99.4%) in the previous study [34]. Additionally, kaurene presented antimicrobial activity against oral pathogens [35] and bisabolene epoxide exhibited an interesting antimicrobial activity [36,37]. In addition to other sesquiterpenes, triterpenes, and hydrocarbons which were previously reported for their antimicrobial activities [38].

*P. gingivalis* is a Gram-negative oral anaerobe causing periodontitis, a severe gum infection, which could lead to tooth breakdown and loss in addition to many other serious complications [5].

In the present work, the frankincense extract exhibited antibacterial activity against *P. gingivalis* clinical isolates with MIC values ranged from 500 to 1000 µg/mL. Some researchers have reported that the frankincense extract has antibacterial activity against different bacterial pathogens such as *Staphylococcus aureus*, *Pseudomonas aeruginosa*, *Propionibacterium acnes*, and *Klebsiella pneumoniae* [39,40]. Al-Hamdoni and Al-Rawi [30] reported that frankincense extract exhibited antibacterial activity against the red complex periodontal pathogens isolated from patients in Mosul, Iraq with MIC value of 2.4 mg/mL. The difference between the MIC values reported in the current study and that of Al-Hamdoni and Al-Rawi [16,30] could be explained by the finding reported by Di Stefano et al. [40]. They noticed that frankincense essential oils derived from three different *Boswellia sacra* cultivars had different MIC values against *Staphylococcus aureus*, *Pseudomonas aeruginosa*, and *Propionibacterium acnes* standard isolates.

We found that the frankincense extract inhibited cellular growth in 58.3% of the tested isolates. To explain the inhibitory effect of the frankincense extract on the growth process of *P. gingivalis* isolates, the leakage of nucleotides from the bacterial cells was measured before and after treatment. We observed that 33.3% of the tested isolates showed a significant increase (*p* < 0.05) in the release of nucleotides from the intracellular compartments which could be due to a decrease in the membrane integrity induced by frankincense extract.

Besides the antibacterial activity of the frankincense extract against *P. gingivalis* isolates, this investigation demonstrated more possible features concerning its antibiofilm activity. In most cases, bacterial cells such as *P. gingivalis* are present mainly embedded in biofilms. Developing biofilms is a strategy adopted by bacteria to help their survival in the host environments [41]. Biofilms are functionally and physiologically different from planktonic bacteria [42]. An important difference is the ability of bacteria embedded in the biofilm to tolerate many antibiotics and antibacterial agents 100–1000 times higher than planktonic bacteria. Another main difference is the protection of bacteria enclosed in the biofilm from the attack by the immune system of the host which enables the bacterial cells to form a persistent infection [43]. Therefore, we studied the effect of the frankincense extract on *P. gingivalis* biofilms using crystal violet assay.

Crystal violet is a basic dye (i.e., produces a positive charge after ionization) that can bind to the negatively charged molecules on the bacterial surface and polysaccharides present in the extracellular matrix [44]. In the current study, the semi-quantitative measurement of the mature biofilms produced by *P. gingivalis* isolates revealed that frankincense extract significantly (*p* < 0.05) inhibited the biofilm formation in five *P. gingivalis* isolates. As crystal violet stains both live and dead bacterial cells, we measured the extracellular polysaccharide production by *P. gingivalis* isolates. Polysaccharide production by bacteria is a crucial part of biofilm formation [45]. Polysaccharides are synthesized by live bacteria only; thus, their level could be related to bacterial viability. We noticed that the polysaccharide production by the tested *P. gingivalis* isolates was significantly reduced after treatment with frankincense extract. The mechanism of reduction of the extracellular polysaccharide matrix of *P. gingivalis* after treatment with frankincense extract still necessitates further future studies.

The bacterial hydrophobic properties are responsible for adhesion and biofilm formation [46,47]. Thus, to investigate the effect of frankincense extract on the hydrophobicity of the tested isolates, we determined the HI before and after treatment. We noticed that 50% of *P. gingivalis* isolates displayed a decrease in their HI after treatment with frankincense extract. This outcome might be a factor contributing to the reduction of biofilm formation by *P. gingivalis* isolates induced by the frankincense extract.

Examination of the biofilm formation by both light microscope and SEM before and after treatment with the frankincense extract confirmed the reduction of biofilm formation by frankincense extract.

Biofilm formation by *P. gingivalis* bacteria is regulated by various genes. Amongst them, the long fimbriae A (FimA) encoded by the *fim*A gene, which plays an important role in *P. gingivalis* adhesion, thus affecting biofilm formation [48]. Arg-gingipain (RgpA) in addition to lysine-specific cysteine proteases (Kgp) are necessary for the maturation and processing of Fim A proteins, therefore supporting *P. gingivalis* adhesion to the host tissues [49]. The heme genes (*hag*A, *hag*B) facilitate the acquirement of heme from red blood cells which are necessary for the growth of *P. gingivalis* bacteria, and they are involved in the bacterial adhesion to the human gingival epithelial cells. Hence *fim*A, *rgp*A, *kgp, hag*A, and *hag*B genes are critical in *P. gingivalis* pathogenicity and biofilm formation. Consequently, we determined the relative expression of these genes in *P. gingivalis* isolates treated with frankincense extract by qRT-PCR. We observed a significant decrease in the gene expression of *fim*A, *hag*A, and *hag*B in 50%, 41.7%, and 50% of the isolates, respectively. There was a non-significant change in the gene expression of *rgp*A and *kgp*.

Therefore, many more in vitro studies should be carried out to demonstrate the activity of the frankincense extract on the different oral bacteria associated with periodontal diseases other than *P. gingivalis* such as *Aggregatibacter actinomycetemcomitans*, *Tannerella forsythia*, *Treponema denticola*, *Fusobacterium nucleatum*, *Campylobacter rectus*, and *Campylobacter gracilis* [2].

## 4. Materials and Methods

### 4.1. Plant Material

Dried frankincense oleoresin was obtained from a verified *Boswellia sacra* tree in Salalah, Oman. It was collected in December 2020. A voucher sample (No. gpfr2020-11) was deposited at the herbarium of Pharmacognosy Department, Faculty of Pharmacy, Tanta University after identification in plant ecology department, faculty of science, Tanta University. Frankincense oleoresin was ground to obtain a fine powder. The extraction was done by cold maceration method using 100 g of powder in 500 mL 100% ethanol for 48 h each at room temperature till exhaustion, then filtered using, Whatman filter paper standard size (5.0 mm diameter), evaporated under reduced pressure then lyophilized to obtain a residue. The yield of extract was 20.88 g.

### 4.2. Chemicals and Cell Lines

All the utilized chemicals in the present study were of the analytical grade and they were purchased from Merck, USA. HSF cell lines were obtained from Nawah Scientific Inc., (Mokatam, Cairo, Egypt).

### 4.3. GC-MS Data Analysis

The oleoresin *extract* was analyzed by a GC-MS (Perkin Elmer model: Clarus 580/560S) with a column (Elite-5MS) of 30 m length, 0.25 mm diameter, and 0.25 m film thickness. The temperature in the column oven was programmed to rise from 50 to 300 °C in 2 °C increments. Electron impact ionization was used to ionize the sample components (EI, 70 eV). The injector was set to 280 °C, while one of the detectors was set to 200 °C. At a rate of 3.0 scans/s, the mass range of 40–1000 *m*/*z* was scanned. In the split injection method, 1.0 μL of pure frankincense oleoresin extract was manually injected into the GC-MS using a Hamilton syringe for total ion chromatographic analysis. The GC-MS takes 48 min to complete. The relative percentage of extract constituents was expressed as a percentage with peak area normalization [50].

### 4.4. Identification of Compounds

Mass spectroscopy was used to identify the bioactive compounds by comparing their retention indices and mass spectra fragmentation patterns to those found in the National Institute of Standards and Technology (NIST) library.

### 4.5. Selection of Patients

The study was carried out on 30 systemically healthy patients of both sexes their ages ranged from 25 to 50 years old, diagnosed with moderate to severe chronic periodontitis (majority stage III grade b periodontitis), Table S2 contains more information about the patients, such as age, sex, stage of periodontitis of each patient. The patients were selected from the clinic of Periodontology, Faculty of Dentistry, Tanta University. All the selected patients were systemically free, non-smoker, and not under any systemic antibiotic before or at time of sampling. If female, they were not lactating or pregnant.

### 4.6. Ethical Statement

This clinical and microbiological in vitro study was conducted according to principles guidelines outlined in the declaration of Helsinki on experimentation involving human subjects. The study protocol and consents are approved by the Research Ethics Committee, Faculty of Pharmacy, Tanta University on 2021, and Clinical Trials.gov ID: NCT: NCT04705714.

### 4.7. Bacterial Isolation

Gingival crevicular fluid samples (GCF) were collected from pockets having ≥5 mm depth and positive bleeding on probing (active disease) with a paper point. The selected site was isolated with sterile cotton rolls, and the supragingival plaque was discarded with sterile cotton pellets. Two sterilized paper points (size # 40) were carefully inserted to the maximum depth of the periodontal pocket and held in position for ten seconds, and then they were placed immediately in one milliliter of transport medium in an Eppendorf tube and another sample from the same site in a test tube contains one milliliter of transport medium. Samples were obtained from the periodontal pockets after removing the supragingival plaque from the teeth to be sampled. Figure S50 shows the method of subgingival plaque sample collection using sterile paper points.

The subgingival plaque samples were then inoculated into 2 mL brain heart infusion (BHI) broth supplemented with 5 μg/mL hemin and 1 μg/mL menadione (vitamin K_1_). They were then diluted and cultured onto blood agar supplemented with 5% sheep blood, hemin (5 μg/mL) and vitamin K_1_ (0.5 μg/mL). These plates were incubated in duplicate for 7–10 days at 37 °C in an anaerobic atmosphere. The bacteria grown were finally selected based on their color, size, and shape. The black-pigmented colonies and Gram-negative rods (when examined microscopically) were examined by a fluorescence test using longwave UV light. The absence of fluorescence distinguishes between *P. gingivalis* and other anaerobic, black-pigmented, Gram-negative rods [51]. The identification of *P. gingivalis* isolates was then confirmed using API 20A (BioMérieux, France). *P. gingivalis* ATCC 33,277 was used as a reference strain.

### 4.8. Antibacterial Screening

It was performed using the agar diffusion method [52]. In brief, bacterial suspension was distributed onto blood agar plate surfaces supplemented with 5% sheep blood, 1% (*v*/*v*) hemin, and 1% (*v*/*v*) vitamin K_1_, then sterilized 6 mm blank filter paper discs were impregnated with 25 µL of frankincense oleoresin extract with different concentrations ranging from 0.5 to 1000 µg/mL. Discs loaded with 10% DMSO were used as negative controls [53,54] and tetracycline disc (30 µg) was used as a positive control. All Petri dishes were anaerobically incubated at 37 °C for 48 h. Inhibition zone diameters were measured and the concentrations of frankincense extract which showed clear zones (inhibition zone) around the discs were regarded to have an inhibitory effect on the tested bacteria.

### 4.9. Determination of MIC Values

The MIC values of frankincense extract against *P. gingivalis* isolates were estimated using the broth microdilution method [8,55,56]. Briefly, cultures of *P. gingivalis* were grown overnight in BHI broth containing hemin (5 µg/mL) and vitamin K_1_ (1 µg/mL). Then, 100 µL of bacteria (diluted first with fresh BHI to obtain an optical density of 0.2 at 660 nm) plus 100 µL of serial dilutions (two-fold) of the frankincense extract (starting from 2000 µg/mL) in BHI were mixed in the wells of the microtitration plate. Each microtitration plate had a negative control well-containing BHI without bacteria and a positive control well containing only bacteria without the extract. After anaerobic incubation at 37 °C for 24 h, bacterial growth was inspected visually. The MIC values were recorded as the lowest concentrations that caused an inhibition of the bacterial growth. All the following experiments were performed before and after treatment of *P. gingivalis* isolates with sub-inhibitory concentrations (0.5 MIC) of the frankincense extract.

### 4.10. Bacterial Growth Curve

*P. gingivalis* isolates were cultured in blood agar plates, supplemented with 5% sheep blood, hemin (5 μg/mL), and vitamin K_1_ (0.5 μg/mL), for 5–7 days under anaerobic conditions at 37 °C [57]. Then, a single colony of each isolate was inoculated into BHI broth, containing hemin (5 µg/mL) and vitamin K_1_ (1 µg/mL), and grown anaerobically for 24 h. The optical density (OD) values at 600 nm were detected every 2 h using UV-VIS spectrophotometer (Shimadzu, Japan), and the growth curves were constructed via plotting the log OD against the sampling time (hrs.).

### 4.11. Measurement of Nucleic Acid Leakage

The release of nucleic acids from the bacterial cells was measured as described previously [58,59]. *P. gingivalis* isolates, at log phase, were centrifuged and the pellets were resuspended in phosphate-buffered saline (PBS), and incubated under anaerobic conditions. The release of cellular nucleic acids was measured at different times of 0, 1, 2, and 4 h. Through measuring the absorbance at 260 nm using an 1800 UV-VIS spectrophotometer (Shimadzu, Japan).

### 4.12. Bacterial Cell Surface Hydrophobicity

The bacterial hydrophobicity was assessed using the hydrocarbon-xylene test [55]. Briefly, *P. gingivalis* isolates were cultured anaerobically and the pellets collected after centrifugation were resuspended in phosphate urea magnesium sulfate buffer (PUM buffer, pH 6.9). Then, volumes of 0.3, 0.9, 1.2, and 1.8 mL of n-hexane were added to 4.8 mL of the bacterial suspension. The two phases were vortexed, the mixtures were left for 2 h, and the aqueous phase was cautiously separated. The absorbance of the tested isolates that remained in the aqueous phase was measured at 540 nm. HI was identified by the following equation:

$$\mathrm{HI}=\frac{A540\;\mathrm{control}-A540\;\mathrm{test}}{A540\;\mathrm{control}}$$

### 4.13. Antibiofilm Activity of Frankincense Extract

It was performed as previously described [60]. Briefly, 100 µL of *P. gingivalis* suspensions were inoculated and cultured into a 96-well microtitration plate at anaerobic conditions at 37 °C. After incubation for 72 h, the non-adherent cells were removed via washing with PBS three times, and they were left to dry for 1 h. The biofilms were stained using a solution of 0.1% crystal violet and the samples were washed thrice with distilled water. For quantitative analysis of biofilm production, 125 µL of 30% acetic acid was added for 15 min and the OD at 492 nm was measured using ELISA Auto Reader (Sunrise Tecan, Austria). The percentage of biofilm reduction was calculated using the formula:

$$\%\;\mathrm{reduction}\;\mathrm{of}\;\mathrm{biofilm}\;\mathrm{formation}=\frac{\mathrm{OD}\;\mathrm{before}\;\mathrm{treatment}-\mathrm{OD}\;\mathrm{after}\;\mathrm{treatment}}{\mathrm{OD}\;\mathrm{before}\;\mathrm{treatment}}\times 100$$

### 4.14. Measurement of the Extracellular Polysaccharides

Polysaccharide quantification assay was performed because polysaccharides are the main component in the formed biofilm and its quantity would affect biofilm formation [45,57]. The polysaccharides found in the extracellular polymeric substance (EPS) of the formed biofilm were estimated using the phenol-sulfuric acid method [57]. In brief, microtitration plates, after 3 days of biofilm formation by *P. gingivalis* isolates, were washed with PBS to remove the free-floating bacteria and air-dried. Then, 40 μL sterile water plus 40 μL 6% phenol solution, followed by 200 μL 97% sulfuric acid were added to each well. The plates were then left at room temperature for 30 min and the quantity of polysaccharides in the formed biofilm were determined by measuring the absorbance at an OD of 490 nm using ELISA Auto Reader (Sunrise Tecan, Austria). We used different glucose concentrations (0, 5, 10, 20, and 100 mg/L) as a standard to convert the OD values to polysaccharide concentrations.

### 4.15. Examination of Biofilm Morphology by Light Microscope and SEM

Examination of biofilm morphology by light microscope and SEM was carried out according to Qi et al. [57]. Briefly, two groups of glass cover slides were flooded with each *P. gingivalis* isolate (before and after treatment with frankincense extract) and they were left for three days under anaerobic conditions to allow the isolates to form biofilms. The first group of the formed biofilms by *P. gingivalis* isolates was visualized by a bright-field microscope (Labomed, America) using 100× magnification after staining with crystal violet. The second group of the formed biofilms was rinsed using PBS and submerged in 2.5% glutaraldehyde solution for 24 h at 4 °C. Then, they were sequentially dehydrated using a series of ethanol; concentrations ranging from 30% to 100%, let to dry, and sputter-coated with gold for examination with SEM (Hitachi, Japan).

### 4.16. qRT-PCR

Relative gene expression of *fim*A, *hag*A, *hag*B, *rgp*A and *kgp* genes was examined using qRT-PCR. The used primers are listed in Table S1 and the 16S rRNA gene was the housekeeping gene or endogenous control [8]. All the experiments were performed three times and the results values were expressed as mean ± standard deviation (SD). The amplification was carried out by Power SYBR^®^ Green master mix (Thermo SCIENTIFIC, Waltham, MA, USA) using Rotor-Gene Q5 plex instrument (Qiagen, Germany). The relative gene expression was determined using the method of 2^−ΔΔCt^ [61] using the untreated isolates as control samples (its expression was set to 1). Changes in the gene expression with ≥2-fold (increased or decreased) were considered to be statistically significant [62].

### 4.17. Cytotoxicity Assay

The SRB assay was used to determine cell viability. In 96-well plates, aliquots of 100 μL cell suspension (5 × 10^3^ cells) were incubated in complete medium for 24 h. Another aliquot of 100 μL media containing the extract at various concentrations was used to treat the cells. After 72 h of the exposure to the extract, the cells were fixed by replacing the medium with 150 μL of 10% trichloroacetic acid (TCA) and incubating for one hour at 4 °C. After removing the TCA solution, the cells were washed five times with distilled water. Aliquots of 70 μL of SRB solution (0.4 percent *w*/*v*) were added and incubated at room temperature for 10 min in the dark. Plates were washed three times with 1% acetic acid and air-dried overnight. The absorbance was measured at 540 nm using a BMG LABTECH^®^- FLUOstar Omega microplate reader (Ortenberg, Germany) after 150 μL of TRIS (10 mM) was added to dissolve protein-bound SRB stain [31,63].

### 4.18. Statistical Analysis

Data were analyzed statistically by one-way analysis of variance (ANOVA) using the SPSS software version 18.0 (IBM, New York, NY, USA). All measurements were performed three times and expressed as mean ± SD. Results with *p-*values lower than 0.05 (*p* < 0.05) were regarded to be significant.

## 5. Conclusions

In the current investigation, the phytoconstituents of the extract were identified using GC/MS technique. A total of 49 compounds were identified. In addition, the frankincense extract displayed antibacterial activity against *P. gingivalis* isolates and this could be attributed to the variety of active compounds identified in this extract. Moreover, it inhibited the growth of *P. gingivalis* bacteria which might be associated with a reduction of the bacterial membrane integrity. On the other hand, the frankincense extract inhibited the biofilm formation by *P. gingivalis* and reduced the expression of genes encoding for virulence factors associated with biofilm formation. This work paid attention to frankincense extract which could be applied to gums as an effective safe antimicrobial agent for periodontitis prevention and treatment. Thus, a promising future may await *Boswellia sacra* Flueck. oleoresin extract in antimicrobial drug discovery. In addition, in vivo future studies should be conducted on the frankincense extract as it may participate in drug discovery.

## Figures and Tables

**Figure 1:**
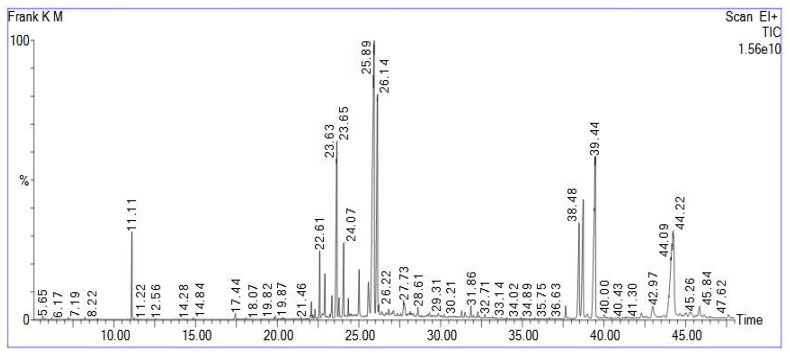
GC/MS total ion chromatogram of the frankincense oleoresin ethanol extract.

**Table 1:** Volatile phytoconstituents identified in the frankincense oleoresin ethanol extract by GC-MS.

Peak No.	Rt (min.)	Name	Peak Area%
1	11.106	Acetic acid, octyl ester	2.073
2	17.443	Dodecanoic acid	0.273
3	22.090	1,3,6,10-Cyclotetradecatetraene,3,7,11-trimethyl-14-(1-methylethyl)-, [*S*-(*E*,*Z*,*E*,*E*)]	0.424
4	22.310	Bicyclo [9.3.1]pentadeca-3,7-dien-12-ol,4,8,12,15,15-pentamethyl-, [1*R*-(1*R*,3*E*,7*E*,11*R* 12*R*)]-	0.256
5	22.605	Cyclohexane, 1-ethenyl-1-methyl-2,4-bis(1-methylethenyl)-	1.831
6	22.770	*n*-Hexadecanoic acid	0.177
7	22.920	1,6,10,14-Hexadecatetraen-3-ol,3,7,11,15-tetramethyl-, (*E*,*E*)	1.337
8	23.346	ç-Elemene	0.658
9	23.651	Kaur-16-ene	7.459
10	23.786	Cycloheptane, 4-methylene-1-methyl-2-(2-methyl-1-propen-1-yl)-1-vinyl-	0.584
11	24.066	Thunbergol	2.380
12	24.351	Aromadendrene oxide-(2)	0.456
13	25.011	1-Heptatriacotanol	1.488
14	25.581	2,6,10,14-Hexadecatetraen-1-ol, 3,7,11,15-tetramethyl-, acetate, (*E*,*E*,*E*)-	1.601
15	25.932	*Trans*-Nerolidyl formate	19.880
16	26.137	Cis-*Z*-α-Bisabolene epoxide	9.410
17	26.222	Androstan-17-one, 3-ethyl-3-hydroxy-,(5à)-	0.175
18	26.417	Butyl 4,7,10,13,16,19-docosahexaenoate	0.165
19	26.827	Vitamin A aldehyde	0.187
20	26.997	i-Propyl 5,8,11,14,17-eicosapentaenoate	0.125
21	27.122	1-Naphthalenepropanol, à-ethenyldecahydro-2-hydroxy-à,2,5,5,8a-pentamethyl-, [1*R*-[1à(*R* *),2á,4aá,8aà]]-	0.280
22	27.732	Cholestan-3-ol,2-methylene-,(3á,5à)-	0.497
23	27.782	2-[4-methyl-6-(2,6,6-trimethylcyclohex-1-enyl) hexa-1,3,5-trienyl] cyclohex-1-en-1-carboxaldehyde	0.482
24	28.057	Docosahexaenoic acid	0.136
25	28.132	Isoaromadendrene epoxide	0.153
26	28.613	Retinol, acetate	0.397
27	29.313	Card-20(22)-enolide, 3,5,14,19-tetrahydroxy-,(3á,5á)-	0.159
28	29.853	9,10-Secocholesta-5,7,10(19)-triene-3,25,26-triol, (3á,5*Z*,7*E*)-	0.175
29	31.289	3-Oxatricyclo [20.8.0.0(7,16)] triaconta-1(22),7(16), 9,13,23,29-hexaene	0.236
30	31.494	Hexadecanoic acid, 2-hydroxy-1-(hydroxymethyl)ethyl ester	0.269
31	31.859	Methyl 2-hydroxy-octadeca-9,12,15-trienoate	0.360
32	32.274	Butyl 6,9,12,15-octadecatetraenoate	0.174
33	37.656	2*H*-Cyclopenta[a]phenanthrene-3,17-dione, 16-(1,3-dimethyl-1*H*-pyrazol-4-ylmethylene)-10,13-dimethyl-1,6,7,8,9,10,11,12,13,14,15,16-dodecahydro-	0.438
34	38.477	Retinol	4.236
35	38.732	2(1H)Naphthalenone, 3,5,6,7,8,8a-hexahydro-4,8a-dimethyl-6-(1-methylethenyl)-	6.204
36	38.977	Prasterone	0.199
37	39.442	Urs-12-en-24-oic acid, 3-oxo-, methyl ester, (+)-7	12.420
38	39.997	9,19-Cycloergost-24(28)-en-3-ol,4,14-dimethyl-, acetate, (3á,4à)	0.157
39	42.283	Oleana-11,13(18)-diene	0.341
40	42.558	Betulin	0.196
41	42.973	Urs-12-ene	1.064
42	43.673	Lanosterol	0.149
43	44.224	9,19-Cyclolanost-24-en-3-ol, acetate, (3a)-(cycloartenol acetate)	13.560
44	44.659	4,4,6a,6b,8a,11,11,14b-Octamethyl-1,4,4a,5,6,6a,6b,7,8,8a,9,10,11,12,12a,14,14a,14b-octadecahydro-2*H*-picen-3-one	0.170
45	45.019	Acetic acid, 3-hydroxy-7-isopropenyl-1,4a-dimethyl-2,3,4,4a,5,6,7,8-octahydronaphthalen-2-yl ester	0.280
46	45.264	α-Amyrin	0.472
47	45.624	Stigmasterol	0.144
48	45.844	2-Oleanen-3-yl acetate, (3à)-	0.822
49	47.630	Lupeol	0.206

**Table 2:** MIC values of frankincense extract against *P. gingivalis* isolates.

Isolate Code	P1	P2	P3	P4	P5	P6	P7	P8	P9	P10	P11	P12
MIC value (µg/mL)	500	1000	1000	1000	500	500	500	1000	1000	500	500	500

**Table 3:** The relative gene expression (mean± SD) for the tested *P. gingivalis* isolates after treatment with frankincense extract.

Isolate Code	Relative Gene Expression *				
	*fim*A	*hag*A	*hag*B	*rgp*A	*Kgp*
P1	**0.1 ± 0.3**	**0.6 ± 0.1**	1.1 ± 0.3	1.1 ± 0.1	1.2 ± 0.2
P2	**0.3 ± 0.1**	**0.3 ± 0.1**	**0.5 ± 0.2**	0.8 ± 0.2	1.0 ± 0.3
P3	1.4 ± 0.0	**0.1 ± 0.1**	**0.3 ± 0.1**	1.2 ± 0.3	1.2 ± 0.1
P4	1.1 ± 0.3	**0.5 ± 0.2**	**0.4 ± 0.1**	1.0 ± 0.2	1.4 ± 0.2
P5	**0.5 ± 0.1**	**0.1 ± 0.2**	**0.4 ± 0.2**	0.8 ± 0.2	1.1 ± 0.2
P6	**0.3 ± 0.2**	1.2 ± 0.1	**0.2 ± 0.09**	0.8 ± 0.1	1.4 ± 0.1
P7	1.2 ± 0.2	1.4 ± 0.1	**0.6 ± 0.1**	1.2 ± 0.2	0.9 ± 0.3
P8	1.4 ± 0.0	1.2 ± 0.4	1.2 ± 0.1	0.8 ± 0.1	1.4 ± 0.0
P9	1.3 ± 0.2	1.0 ± 0.2	1.3 ± 0.2	1.0 ± 0.2	0.8 ± 0.2
P10	**0.2 ± 0.2**	1.2 ± 0.1	1.3 ± 0.1	1.1 ± 0.3	1.2 ± 0.0
P11	1.2 ± 0.0	1.2 ± 0.0	1.2 ± 0.0	1.2 ± 0.1	0.9 ± 0.3
P12	**0.4 ± 0.2**	1.0 ± 0.2	1.0 ± 0.1	1.1 ± 0.3	1.2 ± 0.1

* The bolded values point to a significant decrease in gene expression.

## Data Availability

The authors confirm that the data supporting this study are available within the article [and/or] its Supplementary Materials.

## Supplementary Materials

The following are available online at https://www.mdpi.com/article/10.3390/antibiotics10070859/s1, Figures from S1 to S49: Mass fragmentation pattern of identified compounds. Figure S50 shows the method of subgingival plaque sample collection using sterile paper points. Table S1: Sequences of the oligonucleotides used in qRT–PCR. Table S2: Age, sex, stage of periodontitis of the selected patient.
