# Supplementary material for: Antibacterial Activity of Boswellia sacra Flueck. Oleoresin Extract against Porphyromonas gingivalis Periodontal Pathogen
Source: Antibiotics (Basel). 2021 Jul 15;10(7):859. doi: 10.3390/antibiotics10070859 (PMC8300764; doi:10.3390/antibiotics10070859)
Supplement: Supplementary file 1 [file antibiotics-10-00859-s001.zip › antibiotics-1284685-supplementary.pdf]

Supporting Materials

Antibacterial Activity of *Boswellia sacra* Flueck. Oleoresin Extract against *Porphyromonas gingivalis* Periodontal Pathogen

Nashwah G. M. Attallah [1,2,†], Walaa A. Negm [3,*,†], Engy Elekhnawy [4,*], Najla Altwaijry [1], Elshaymaa I. Elmongy [1,5,*], Thanaa A. El-Masry [6], Eman A. Alturki [7], Doaa A. Yousef [8] and Malak Y. Shoukheba [8]

[1] Pharmaceutical Sciences Department, College of Pharmacy, Princess Nourah bint Abdulrahman University, 84428 Riyadh, Saudi Arabia; ngmohamed@pnu.edu.sa (N.G.M.A.); naaltwaijry@pnu.edu.sa (N.A.)
[2] Egyptian Drug Authority (EDA), 8655 Giza, Egypt (previously NODCAR)
[3] Pharmacognosy Department, Faculty of Pharmacy, Tanta University, 31527 Tanta, Egypt
[4] Pharmaceutical Microbiology Department, Faculty of Pharmacy, Tanta University, 31527 Tanta, Egypt
[5] Pharmaceutical Chemistry Department, Faculty of Pharmacy, Helwan University, 11795 Helwan, Egypt
[6] Pharmacology Department, Faculty of Pharmacy, Tanta University, 31527 Tanta, Egypt; thanaa.elmasri@pharm.tanta.edu.eg (T.A.E.-M)
[7] Pharmacognosy Department, College of Pharmacy, King Saud University, 84428 Riyadh, Saudi Arabia; emalturki@ksu.edu.sa
[8] Oral Medicine, Periodontology, Oral Diagnosis, and Radiology Department, Faculty of Dentistry, Tanta University, 31527 Tanta, Egypt; doaa.bayoumi@dent.tanta.edu.eg (D.A.Y.); malak.mohamed@dent.tanta.edu.eg (M.Y.S.)
* Correspondence: walaa.negm@pharm.tanta.edu.eg (W.A.N.); engy.ali@pharm.tanta.edu.eg (E.E.); eielmongy@pnu.edu.sa (E.I.E.)
† These authors contributed equally to this work.

Table S1: Sequences of the oligonucleotides used in qRT–PCR.

Gene	Direction*	Sequence
16S rRNA	F	5′-TGTAGATGACTGATGGTGAAA-3′
	R	5′ -ACTGTTAGCAACTACCGATGT-3′
*fim*A	F	5′-CAGCAGGAAGCCATCAAATC-3′
	R	5′ -CAGTCAGTTCAGTTGTCAAT-3′
*hag*A	F	5′-ACAGCATCAGCCGATATTCC-3′
	R	5′-CGAATTCATTGCCACCTTCT-3′
*hag*B	F	5′-TGTCGCACGGCAAATATCGCTAAAC-3′
	R	5′-CTGGCTGTCCTCGTCGAAAGCATAC-3′
*rgp*A	F	5′-GCCGAGATTGTTCTTGAAGC-3′
	R	5′-AGGAGCAGCAATTGCAAAG-3′
Kgp	F	5′-AGCTGACAAAGGTGGAGACCAAAGG-3′
	R	5′– TGTGGCATGAGTTTTTCGGAACCGT-3′

*F: forward, R: reverse

Table S2: Age, sex, stage of periodontitis of the selected patient

Patient number	Age	Sex	Stage of periodontitis
**1**	30	Male	Stage III grade B
**2**	40	Female	Stage III grade B
**3**	30	Male	Stage III grade B
**4**	34	Male	Stage III grade B
**5**	32	Male	Stage III grade B
**6**	45	Female	Stage III grade B
**7**	49	Female	Stage III grade B
**8**	50	Male	Stage III grade B
**9**	45	Female	Stage III grade B
**10**	34	Female	Stage III grade A- B
**11**	44	Female	Stage III grade B-C
**12**	42	female	Stage III grade B-C
**13**	25	Male	Stage III grade B
**14**	25	Male	Stage III grade B
**15**	27	Female	Stage III grade B
**16**	33	Male	Stage III grade B
**17**	25	Male	Stage III grade A-B
**18**	39	Male	Stage III grade B
**19**	45	Female	Stage III grade B
**20**	46	Male	Stage III grade B
**21**	47	Female	Stage III grade B
**22**	45	Female	Stage III grade B
**23**	40	Male	Stage III grade B
**24**	24	Male	Stage III grade B
**25**	38	Female	Stage III grade B
**26**	42	Female	Stage III grade B
**27**	46	Male	Stage III grade B
**28**	30	male	Stage III grade B
**29**	33	female	Stage III grade B
**30**	29	male	Stage III grade B

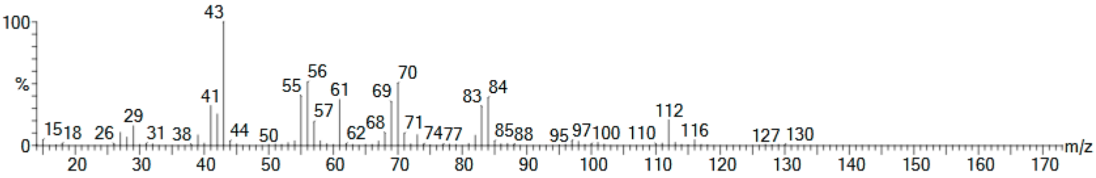

Figure S1 Mass fragmentation pattern of acetic acid, octyl ester

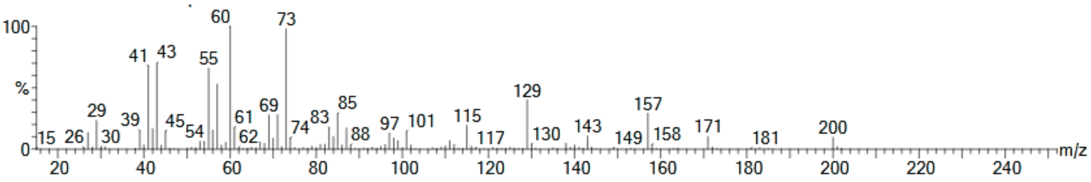

Figure S2 Mass fragmentation pattern of dodecanoic acid

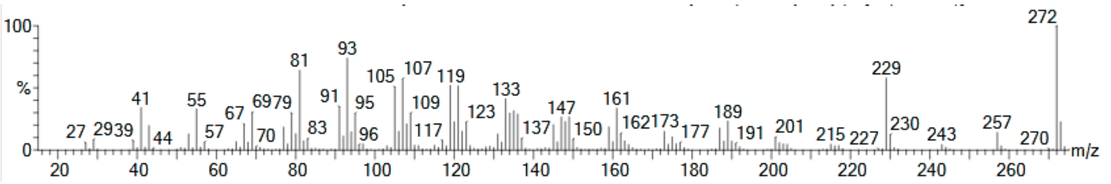

Figure S3 Mass fragmentation pattern of ,3,6,10-cyclotetradecatetraene, 3,7,11-trimethyl-14-(1-methylethyl)-, [S-(E,Z,E,E)]

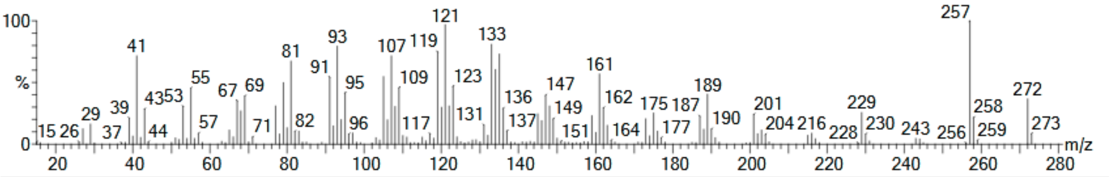

Figure S4 Mass fragmentation pattern of Bicyclo [9.3.1] pentadeca-3,7-dien-12-ol, 4,8,12,15,15-pentamethyl-, [1R-(1R*,3E,7E,11R*,12R*)]

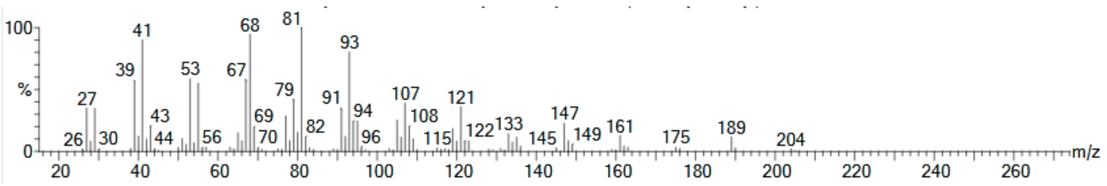

Figure S5 Mass fragmentation pattern of cyclohexane, 1-ethenyl-1-methyl-2,4-bis(1-methylethenyl)

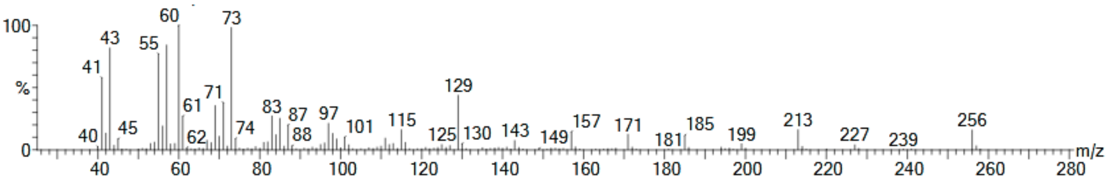

Figure S6 Mass fragmentation pattern of n-hexadecanoic acid

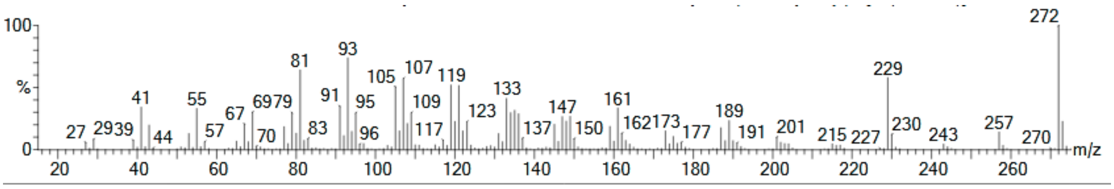

Figure S7 Mass fragmentation pattern of 1,6,10,14-hexadecatetraen-3-ol, 3,7,11,15-tetramethyl-, (E, E)

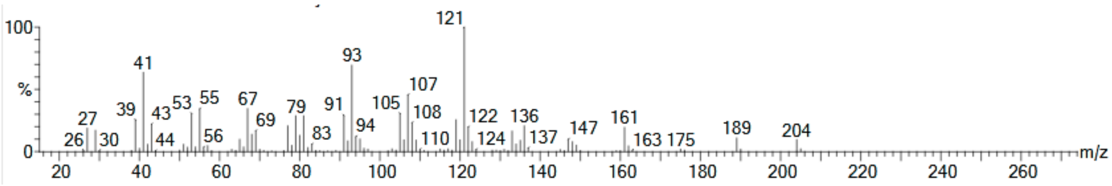

Figure S8 Mass fragmentation pattern of ç-elemene

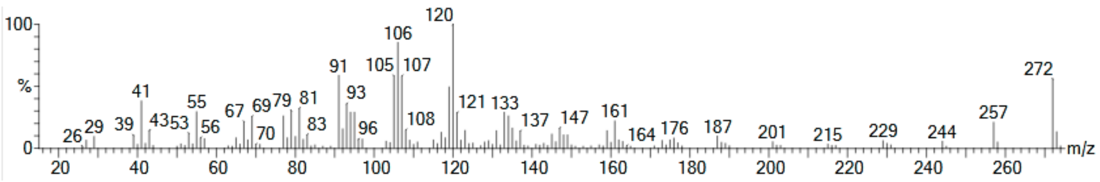

Figure S9 Mass fragmentation pattern of kaur-16-ene

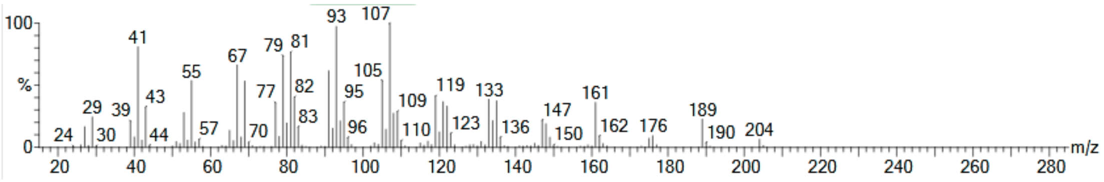

Figure S10 Mass fragmentation pattern of cycloheptane, 4-methylene-1-methyl-2-(2-methyl-1-propen-1-yl)-1-vinyl-

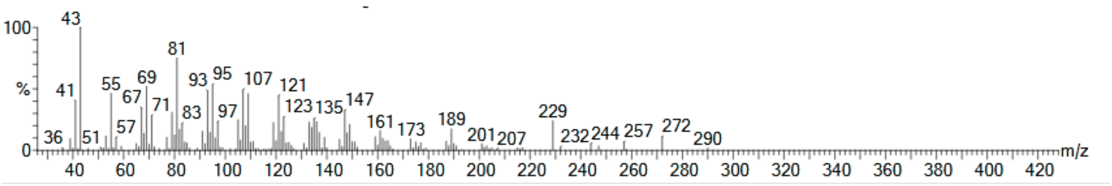

Figure S11 Mass fragmentation pattern of thunbergol

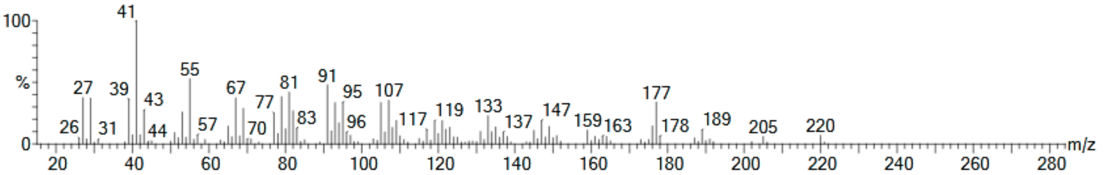

Figure S12 Mass fragmentation pattern of aromadendrene oxide-(2)

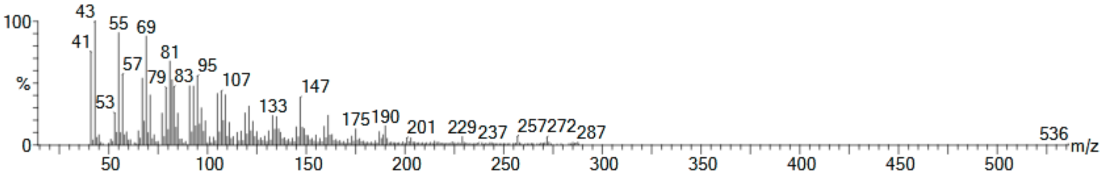

Figure S13 Mass fragmentation pattern of 1-Heptatriacotanol

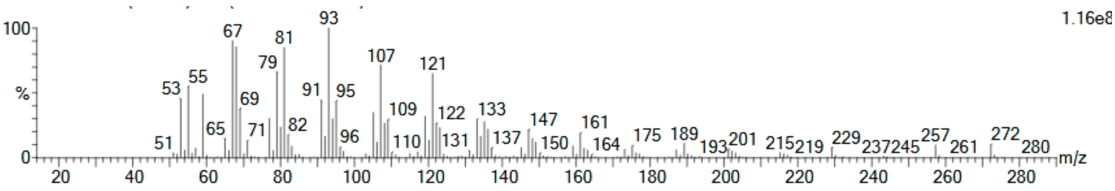

Figure S14 Mass fragmentation pattern of 2,6,10,14-Hexadecatetraen-1-ol, 3,7,11,15-tetramethyl-, acetate, (E,E,E)-

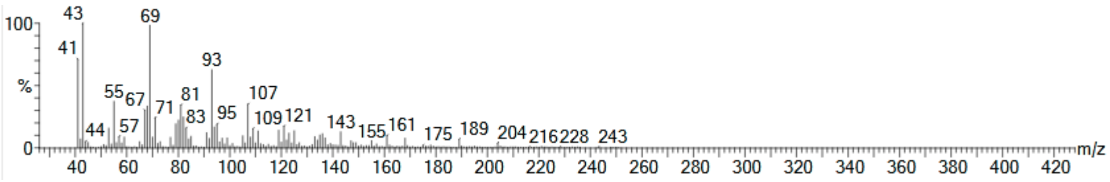

Figure S15 Mass fragmentation pattern of *trans*-Nerolidyl formate

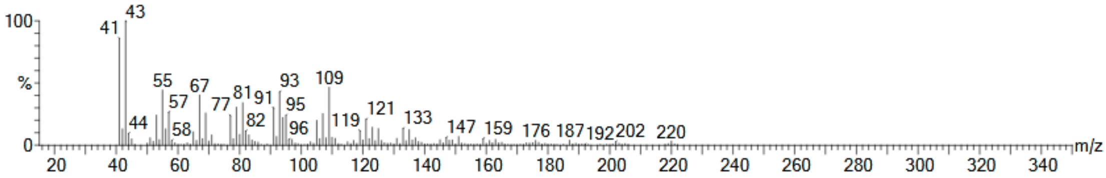

Figure S16 Mass fragmentation pattern of cis-Z-à-Bisabolene epoxide

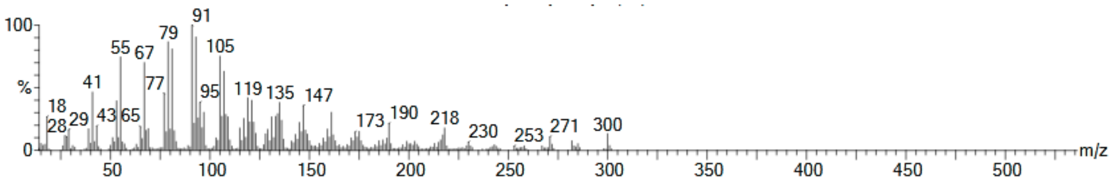

Figure S17 Mass fragmentation pattern of androstan-17-one, 3-ethyl-3-hydroxy-, (5à)-

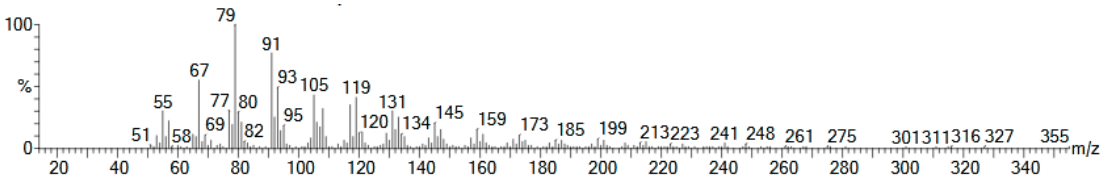

Figure S18 Mass fragmentation pattern of butyl 4,7,10,13,16,19-docosahexaenoate

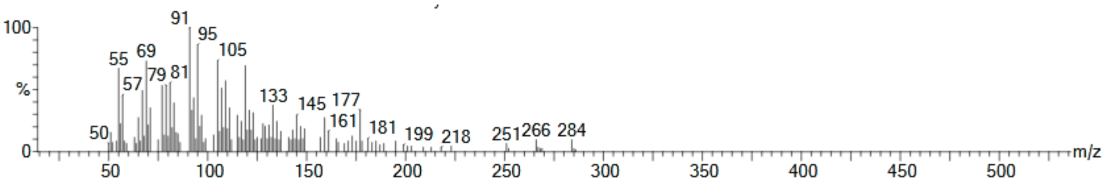

Figure S19 Mass fragmentation pattern of Vitamin A aldehyde

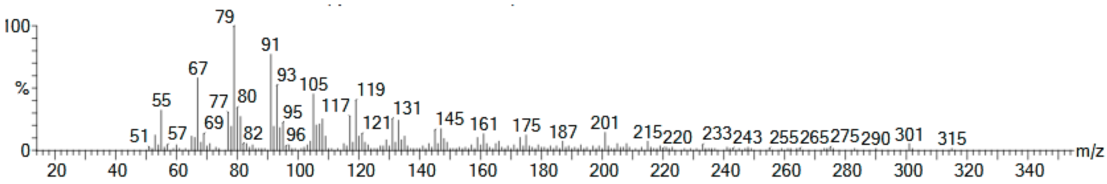

Figure S20 Mass fragmentation pattern of *i*-propyl 5,8,11,14,17-eicosapentaenoate

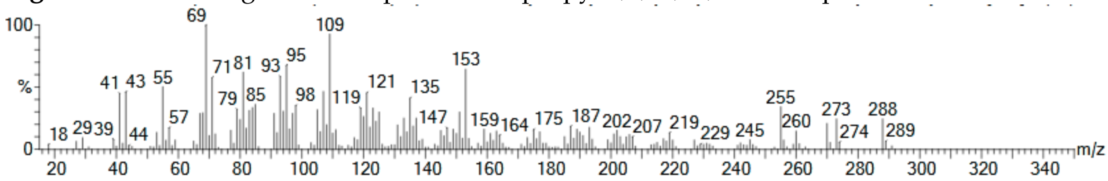

Figure S21 Mass fragmentation pattern of 1-Naphthalenepropanol, à-ethenyldecahydro-2-hydroxy-à,2,5,5,8a-pentamethyl-, [1R-[1à(R*),2á,4aá,8aà]]-

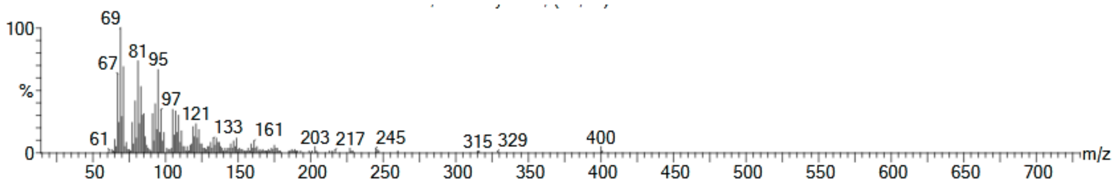

Figure S22 Mass fragmentation pattern of Cholestan-3-ol, 2-methylene-, (3á,5à)-

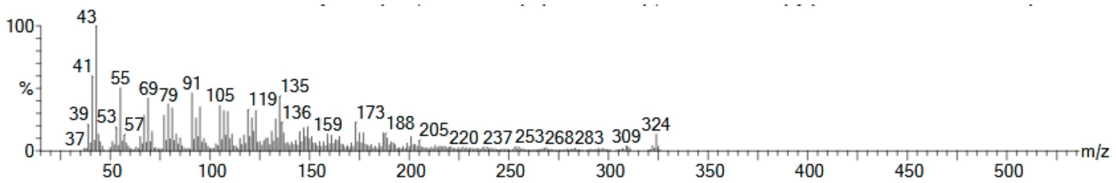

Figure S23 Mass fragmentation pattern of 2-[4-methyl-6-(2,6,6-trimethylcyclohex-1-enyl) hexa-1,3,5-trienyl] cyclohex-1-en-1-carboxaldehyde

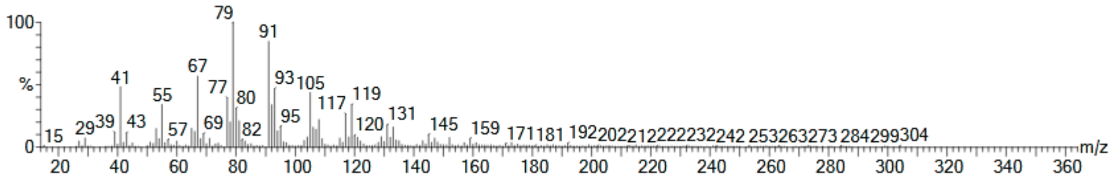

Figure S24 Mass fragmentation pattern of docosahexaenoic acid

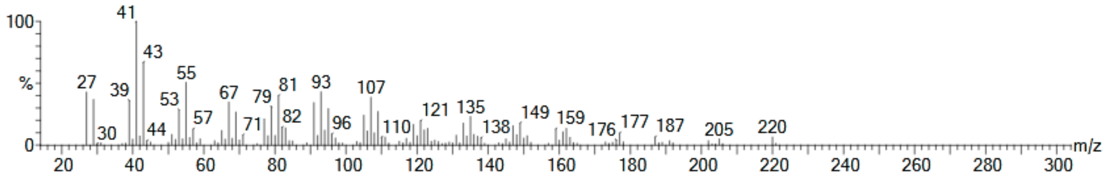

Figure S 25 Mass fragmentation pattern of isoaromadendrene epoxide

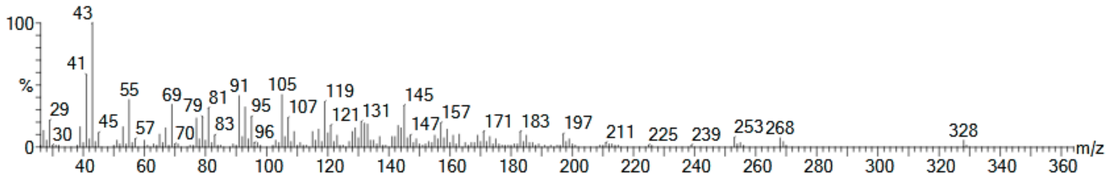

Figure S26 Mass fragmentation pattern of retinol, acetate

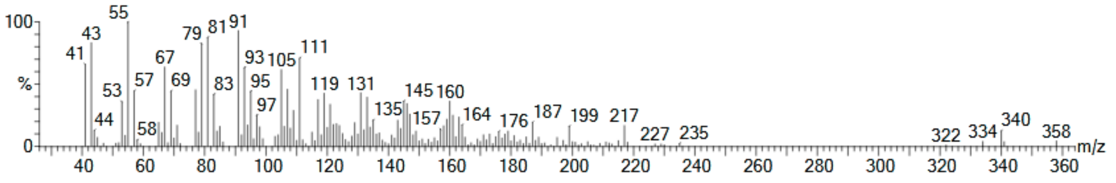

Figure S27 Mass fragmentation pattern of card-20(22)-enolide, 3,5,14,19-tetrahydroxy-, (3á,5á)-

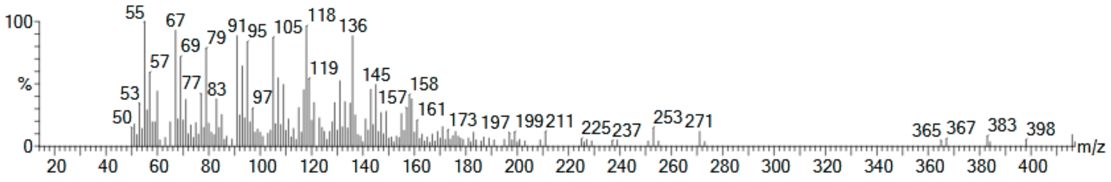

Figure S28 Mass fragmentation pattern of 9,10-secocholesta-5,7,10(19)-triene-3,25,26-triol, (3á,5Z,7E)-

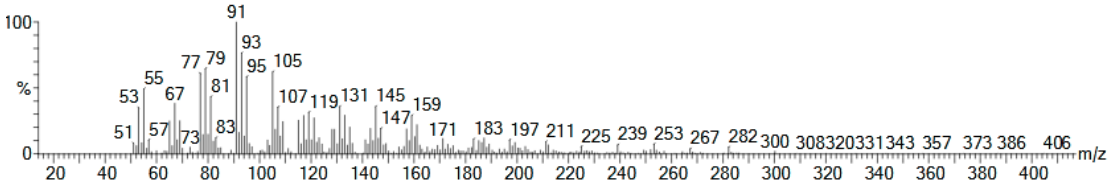

Figure S29 Mass fragmentation pattern of 3-oxatricyclo [20.8.0.0(7,16)] triaconta-1(22),7(16), 9,13,23,29-hexaene

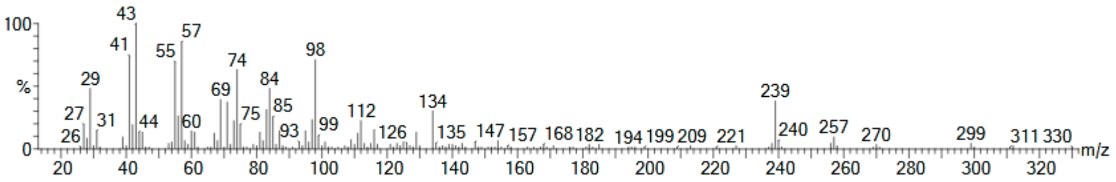

Figure S30 Mass fragmentation pattern of hexadecanoic acid, 2-hydroxy-1-(hydroxymethyl)ethyl ester

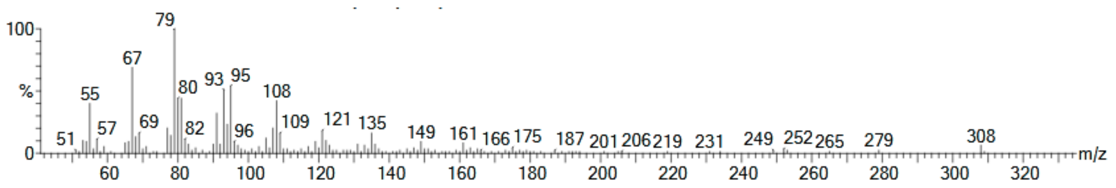

Figure S31 Mass fragmentation pattern of methyl 2-hydroxy-octadeca-9,12,15-trienoate

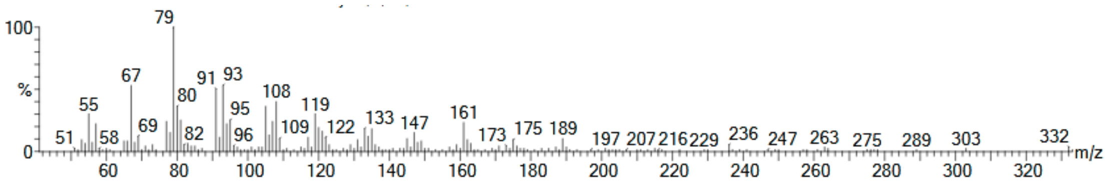

Figure S32 Mass fragmentation pattern of butyl 6,9,12,15-octadecatetraenoate

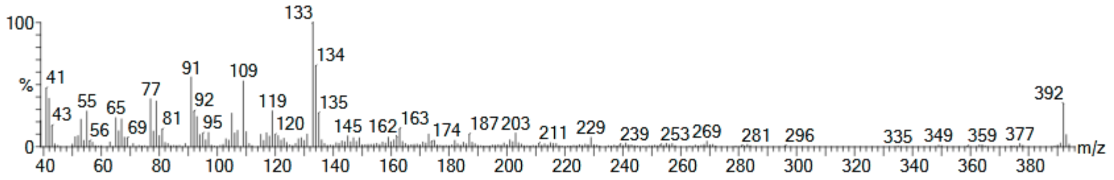

Figure S33 Mass fragmentation pattern of 2H-cyclopenta[a]phenanthrene-3,17-dione, 16-(1,3-dimethyl-1H-pyrazol-4-ylmethylene)-10,13-dimethyl-1,6,7,8,9,10,11,12,13,14,15,16-dodecahydro-

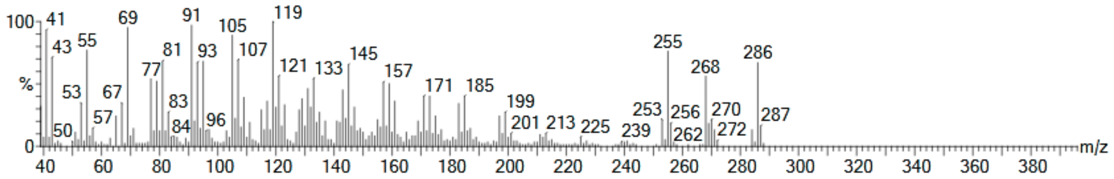

Figure S34 Mass fragmentation pattern of retinol

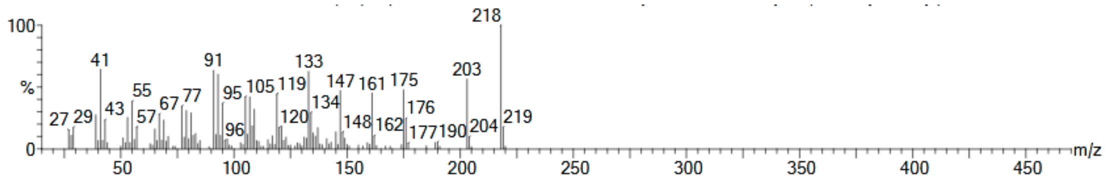

Figure S35 Mass fragmentation pattern of 2(1H) Naphthalenone, 3,5,6,7,8,8a-hexahydro-4,8a-dimethyl-6-(1-methylethenyl)-

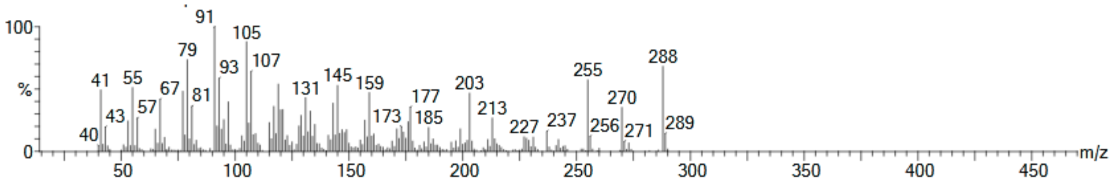

Figure S36 Mass fragmentation pattern of prasterone

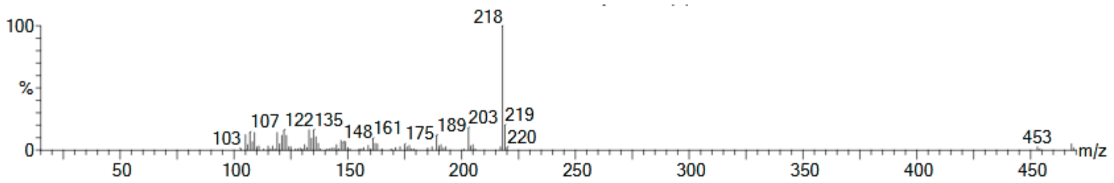

Figure S37 Mass fragmentation pattern of urs-12-en-24-oic acid, 3-oxo-, methyl ester, (+)-7

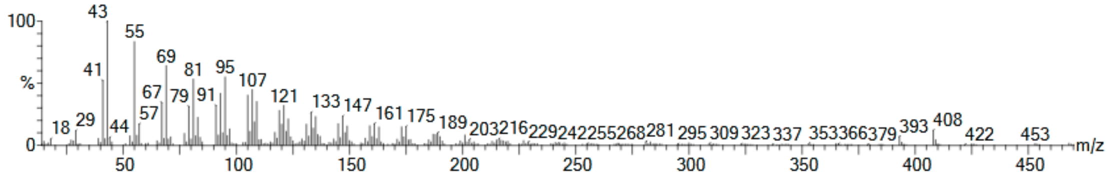

Figure S38 Mass fragmentation pattern of 9,19-cycloergost-24(28)-en-3-ol, 4,14-dimethyl-, acetate, (3á,4à)

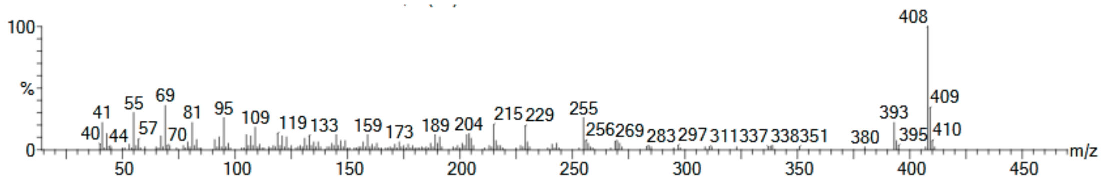

Figure S39 Mass fragmentation pattern of oleana-11,13(18)-diene

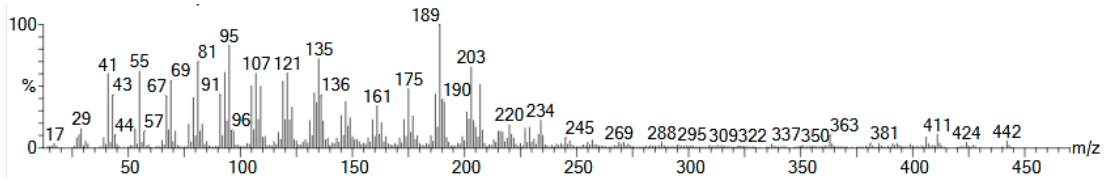

Figure S40 Mass fragmentation pattern of betulin

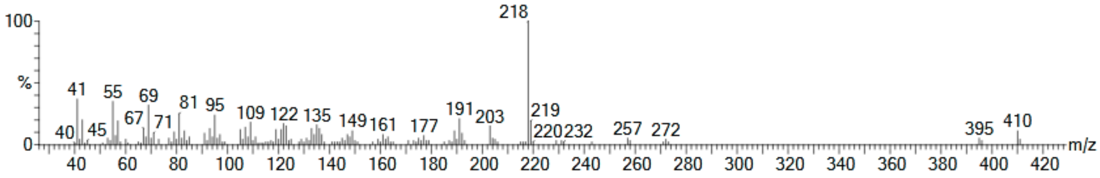

Figure S41 Mass fragmentation pattern of urs-12-ene

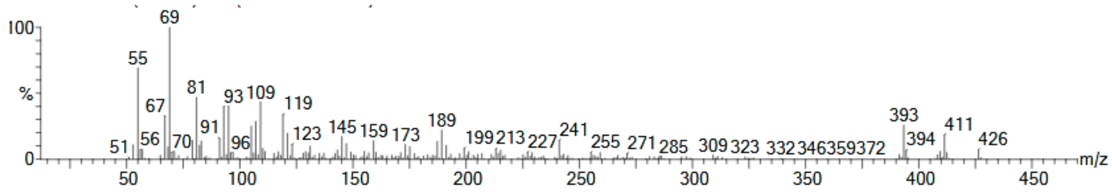

Figure S42 Mass fragmentation pattern of lanosterol

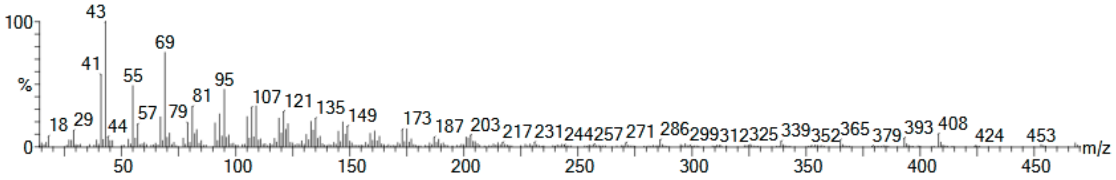

Figure S43 Mass fragmentation pattern of 9,19-cyclolanost-24-en-3-ol, acetate, (3a)-

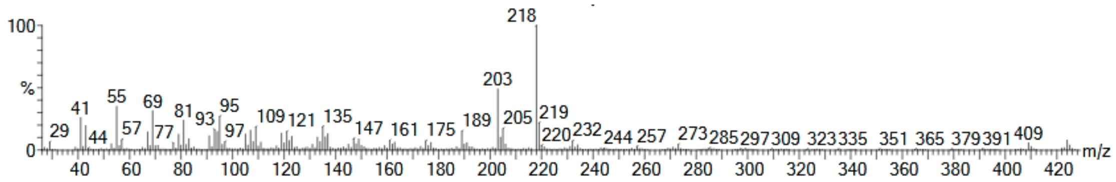

Figure S44 Mass fragmentation pattern of 4,4,6a,6b,8a,11,11,14b-octamethyl-1,4,4a,5,6,6a,6b,7,8,8a,9,10,11,12,12a,14,14a,14b-octadecahydro-2H-picen-3-one

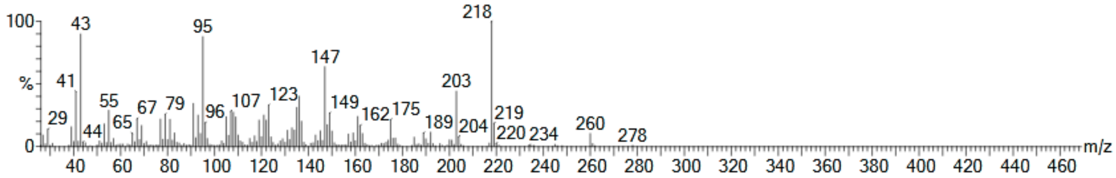

Figure S45 Mass fragmentation pattern of acetic acid, 3-hydroxy-7-isopropenyl-1,4a-dimethyl-2,3,4,4a,5,6,7,8-octahydronaphthalen-2-yl ester

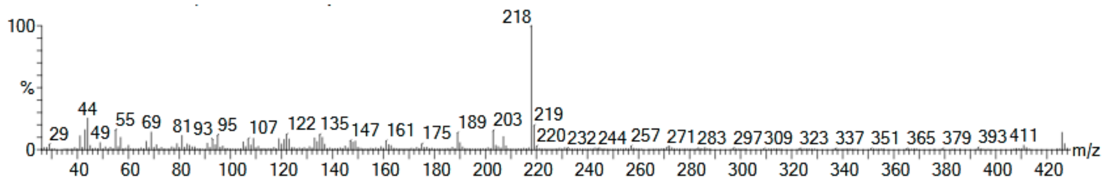

Figure S46 Mass fragmentation pattern of α-amyrin

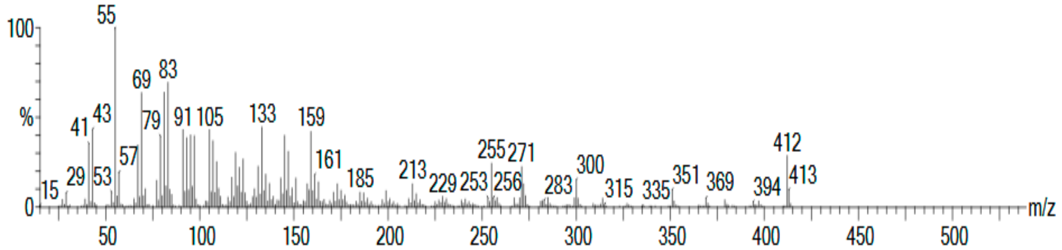

Figure S47 Mass fragmentation pattern of stigmasterol

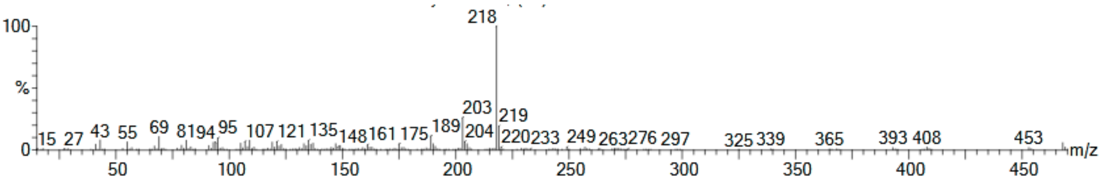

Figure S48 Mass fragmentation pattern of 2-oleanen-3-yl acetate, (3à)-

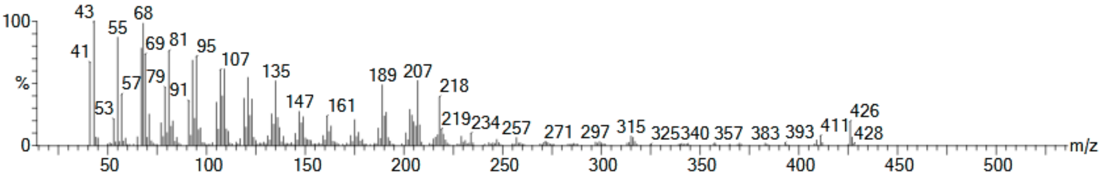

Figure S49 Mass fragmentation pattern of lupeol

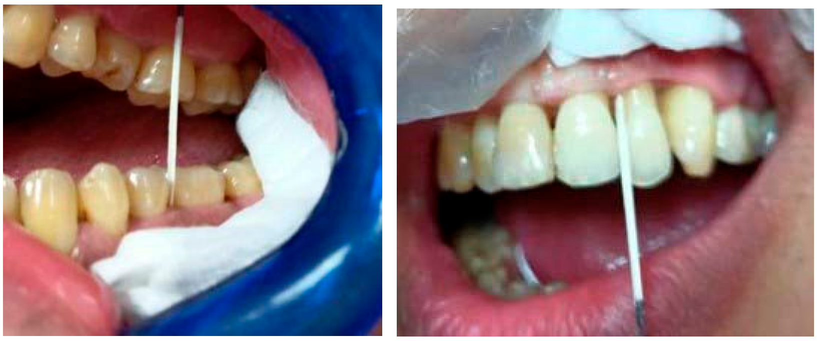

Figure S50 shows the method of subgingival plaque sample collection using sterile paper points.
